# Popular cultural representations of postfeminist religiosity in the International Christian Fellowship: an analysis of the “Ladies Lounge 2021” webpage

**DOI:** 10.1007/s41682-021-00077-x

**Published:** 2021-09-27

**Authors:** Maren Freudenberg, Dunja Sharbat Dar

**Affiliations:** Centrum für Religionswissenschaftliche Studien, Ruhr-Universität Bochum, Universitätsstr. 90a, 44789 Bochum, Germany

**Keywords:** Femininity, Evangelicalism, Gender, Religion, Representations, Mediatization, Popular culture, Postfeminism, International Christian Fellowship, Weiblichkeit, Evangelikalismus, Gender, Religion, Repräsentation, Mediatisierung, Populärkultur, Postfeminismus, International Christian Fellowship

## Abstract

Femininity and female gender roles in conservative religious environments are highly disputed topics both within communities of faith and in sociological discourse. In light of social transformations of gender perceptions in the past decades, conservative Christians have had to reevaluate traditional understandings of womanhood in societies that have become steeped in popular culture and thoroughly mediatized. Taking this development as a point of departure, this article examines how femininity is represented in the International Christian Fellowship, particularly on its “Ladies Lounge” webpage. Advertising an annual event geared exclusively towards women, the website’s landing page contains images and text that we examine by means of visual and textual sequence analysis. Our research results reveal that women are depicted as sensually attractive and self-confidently professional while at the same time being relegated to an exclusively female sphere within (but not beyond) which they wield authority and influence. As such, femininity is represented as self-empowering, but only within a specific, postfeminist framework. This ambivalent depiction of women’s agency challenges conservative Evangelical values at the same time as it affirms them. In this sense, the study contributes the growing body of literature on gender and Evangelicalism.

## Abstract (de)

Weiblichkeit und weibliche Genderrollen in konservativen religiösen Kontexten sind häufig debattierte Themen sowohl innerhalb religiöser Gemeinden als auch im soziologischen Diskurs. In Anbetracht sozialer Transformationen von Genderwahrnehmungen in den vergangenen Jahrzehnten sehen sich konservative Christ*innen gezwungen, traditionelle Verständnisse von Weiblichkeit in Gesellschaften zu reevaluieren, die durchweg mediatisiert und von Populärkultur geprägt sind. Der Beitrag nimmt diesen Prozess als Ausgangspunkt, um zu untersuchen, wie Weiblichkeit im International Christian Fellowship repräsentiert wird, speziell auf der Startseite der „Ladies Lounge“ Webseite. Diese bewirbt einen jährlichen Event, der sich ausschließlich an Frauen richtet; die Startseite beinhaltet Bilder und Text, die wir mit Hilfe von sequenzanalytischen Methoden untersuchen. Unsere Ergebnisse zeigen, dass Frauen als sinnlich-attraktiv und selbstbewusst-professionell dargestellt und gleichzeitig einer exklusiv weiblichen Sphäre zugeordnet werden, innerhalb derer sie Autorität und Einfluss besitzen (aber nicht darüber hinaus). Somit wird Weiblichkeit als selbstermächtigend dargestellt, allerdings nur innerhalb eines spezifischen, postfeministischen Rahmens. Diese ambivalente Repräsentation weiblicher *agency* stellt konservativ-evangelikale Werte einerseits auf den Kopf und bestätigt sie andererseits paradoxerweise. In diesem Sinne stellt der Artikel einen Beitrag zur wachsenden Literatur zu Gender und Evangelikalismus dar.

## Introduction

In this article, we are concerned with the ways in which femininity is represented in images and texts on conservative Christian websites. Drawing from gender studies, discourses on postfeminism, as well as on research on women in Evangelicalism, which are outlined in the following section, we use visual and textual sequence analysis (Betz and Kirchner 2016; Oevermann et al. 1979), briefly introduced in the third section, to analyze a range of images and textual components found on the landing page of the “Ladies Lounge 2021” website (fourth section). The Ladies Lounge is an annual event geared towards women that is hosted by the International Christian Fellowship (ICF), a neo-conservative^1^ Evangelical organization based in Switzerland with congregations all over Europe. We are interested in showing how femininity and religion are mediatized by drawing from popular culture on the Ladies Lounge landing page, and how the gender binary is reproduced in images and texts in subtle yet distinct ways. We argue that the representation of femininity on the Ladies Lounge landing page—and, by extension, within the ICF—is more than a mere blurring of boundaries between religion and popular culture (in the sense of *Entgrenzung*). On the contrary, it should be understood as an act of religious marking (*Markierung*) by way of using popular culture to subtly communicate conservative religious values and societal worldviews. Put differently, webpages such as the one analyzed below reveal the manifold ways in which religion and popular culture enter into dialogue (Forbes and Mahan 2000) and the range of new meanings that emerge in the process.

The International Christian Fellowship (ICF) was founded in Zurich, Switzerland in 1990 and has been headed by its current senior co-pastors, Leo and Susanne Bigger, since 1996 (Friess 2009, p. 3, 2004, p. 23). Because there is no formal membership, no statistics exist on the number of adherents or size of individual congregations (Walthert 2010, p. 245). This is typical of conservative American Evangelicalism and particularly the church growth movement (for an emic perspective, see Wagner 2012), the strategies of which the ICF adopted under the leadership of Leo and Susanne Bigger. Müller notes that two U.S. megachurches, Willow Creek Community Church in Chicago and Saddleback Church in southern California, function as role models for the ICF (Müller 2015, p. 73), while Walthert also lists the Vineyard^2^ as a model that the ICF orients itself towards (2010, p. 246; see also Kunz 2004, p. 19). Its organizational structure, worship culture, and congregational expansion mirror Willow Creek, Saddleback, and Vineyard USA alike.

In August 2021, ICF lists 78 congregations and one “Online Church” worldwide^3^. Worship at the ICF is “seeker-oriented”, a trait adopted from Willow Creek: it caters to young people searching for different forms of spirituality and faith community (Kunz 2004, p. 18) by offering worship with clear popular cultural elements, including contemporary Christian music performed by a band on stage and up-to-date multimedia technology, for instance professional light and sound shows (Friess 2004, p. 25f.). Its small group activities are organized not only thematically, but also by age and gender as structuring categories (Friess 2009, p. 7). These modern elements notwithstanding, the faith convictions and social mores propagated at the ICF are decidedly conservative: the Bible, as the inspired word of God, is the main point of reference in everyday life; salvation is available only to committed believers; a binary worldview, dividing the world into good and evil forces, is adhered to; traditional gender roles, a conservative sexual morality and other antifeminist structures are upheld while homosexual behavior and consuming pornography is considered sinful (Friess 2009, p. 4f., 2004, p. 24, 28). The ICF has been criticized for the pressure it allegedly puts on adherents to be involved in congregations, donate money, and withdraw from their families (Friess 2009, p. 8ff).

The analysis to follow will focus on the online representations of femininity in the framework of a specific ICF format: The Ladies Lounge, an annual event geared towards female members that consists, among other elements, of motivational speeches by popular female pastors, including the ICF’s own senior co-pastor Susanna Bigger and other congregational leaders; large worship gatherings; and opportunities for women to network and connect informally. It is important to point out that this article is not concerned with the Ladies Lounge as an event, but more specifically with the way femininity is represented on the landing page of the Ladies Lounge website. The ICF offers a variety of gender-differentiating formats to attract newcomers, and the range of activities varies from one congregation to the next. It is typically broader for women, or “Ladies,” as they are addressed on most websites, than for men^4^. We chose to analyze the Ladies Lounge landing page as it prominently advertised on the ICF’s main website^5^ as the largest (and, with ticket prices in 2021 at 130 Euros, the most expensive) ICF event geared towards women.

The next section provides a theoretical overview of gender and postfeminism in Evangelical culture and is followed by an introduction to our methodological framework, which centers on visual sequence analysis (Betz and Kirchner 2016). Then, the analysis section presents the most important research results, namely that through its imagery and composition, the Ladies Lounge 2021 webpage communicates womanhood as linked to two central themes: femininity is associated with nature, a stylized yet modest concept of beauty, sensuality, and shelter, on the one hand, and with competence, leadership, self-confidence, and professionalism, on the other. We will argue that both themes additionally address the tension between inclusivity and exclusivity as a core characteristic of femininity in the Ladies Lounge. The contribution closes with a brief concluding outlook.

## Theoretical backdrop: gender and postfeminism in evangelical culture

Gender has long been identified as a “primary category that suffuses all aspects of life” (Juschka 2016, p. 138). Its nature of being socially constructed, especially along the binary division of masculine/feminine, and the fact that it directly influences our perception of gender at the same time as it serves as to actively reproduce this perception, is firmly established in scholarship (e.g., Kessler and McKenna 1978; see also Winkel 2018; Woodhead 2007; Gildemeister 2008; Wetterer 2008, among many others). This becomes very apparent not only but especially in the realm of popular culture, by which we broadly understand, following Hubert Knoblauch (2009), a shared communicative code that enables interaction between very diverse societal groups. As we will see in the analysis to follow, the ICF’s Ladies Lounge casts women as belonging to the informal, sensual, nurturing spheres of everyday life, thus on the one hand reproducing gender roles that developed starting in the early nineteenth century and established women as married and organizing home life while seeking new forms of religious fulfillment and participation (e.g. Winkel 2018; Williams 1991, 1993; Leidner 1991, 1993; see also Gildemeister 2008; Gildemeister and Robert 1999; Wetterer 1995). On the other hand, as we will see, representations of femininity in the Ladies Lounge also emphasize professionalism and self-confident empowerment, thereby drawing from postfeminism and the neoliberal logic alike (McRobbie 2009; Gill 2007; Brown 2006).

Postfeminism emerged as a critical reaction to the feminist gains of the 1970s and 80s, including first and foremost the assertion of gender as socially constructed instead of a ‘natural difference,’ presenting these as outdated norms and values represented by ‘older’ generations and denying them continued validity. Angela McRobbie has shown a routine disparagement and “ritualistic denunciation” (2004, p. 258) of feminism continuously communicated in the media and implicitly expected of women of all ages in what she has called the “double entanglement” of postfeminism: “the co-existence of neo-conservative values in relation to gender, sexuality and family life […] with processes of liberalisation in regard to choice and diversity in sexual, domestic and kinship relations” (2004, p. 255f.). In a nutshell, in the late modern framework of the individual becoming untethered from the restrictions of tradition and responsible for shaping his or her own biography (see Beck 1986,6), postfeminists consciously *choose* to adhere to neo-conservative values, thereby disidentifying with progressive, feminist approaches of the past as supposedly obsolete. McRobbie discusses sexist advertisements as well as the “ambitious ‘TV Blonde’” (2004, p. 257), but also notes “gentler” versions of feminist critique, such as the novel and movie *Bridget Jones’ Diary*, to highlight how postfeminism is deeply entangled with media, popular culture, individualism, and subjectivity. In a similar vein, Aune and Holyoak define postfeminism as “a depoliticized celebration of women’s perceived social and economic emancipation fused with an unproblematic sexualized femininity” (2018, p. 187), pointing out that postfeminism must be viewed as distinct from third-wave feminism, with which it is sometimes conflated.

In her article on postfeminism and media culture (2007), Rosalind Gill discusses several elements of what she calls the “sensibility” of postfeminism that she argues are required to label a text or other media product as “postfeminist”. In the context of the media product analyzed below, the elements of individualism, choice, empowerment, self-surveillance, and discipline are particularly pertinent. Gill argues that the idea of “being oneself” and “pleasing oneself” are “central to the postfeminist sensibility that suffuses contemporary Western media culture,” (2007, p. 153), deeply resonating with themes of empowerment and taking control that can be found in shows such as *Sex and the City* and *Desperate Housewives*, but also makeover shows and, of course, advertisement. The narrative of individualism weaved into such media discourses frames every aspect of life through personal choice and self-determination, shifting the emphasis away from politicization, as a way to publicly tackle larger societal issues, towards claiming one’s right to simply “feel good.” As we will see in the analysis below, postfeminist media culture depicts women as entirely liberated from inequality, completely disregarding how socially constructed and mass-mediated norms of femininity and beauty ideals are objectivated and internalized (Berger and Luckmann 1967) to act back upon the neoliberal subject as “reality”. If life is rendered meaningful through the lens of personal choice and autonomy, seemingly unconstrained by power imbalances, then self-surveillance and personal discipline are key to maintaining this perspective: being “feminine” requires both exterior and interior work, i.e., shaping one’s body and looks as well as one’s psyche. Gill aptly observes that, “in an extraordinary sleight of hand, this labour must be understood nevertheless as ‘fun,’ ‘pampering’ or ‘self-indulgence’ and must *never* be disclosed” (2007, p. 155; emphasis in original).

Popular cultural as well as religious representations of femininity have long been a point of departure for analyzing gender differences^7^. Depictions of female bodies have always been and continue to be central for intra- and inter-religious discourses on heteronormative gender distinctions, reproducing these distinctions in this very process (Lanwerd 2008, p. 211f.). For instance, in their study of a women-only ministry named “God Chicks” in the Oasis Christian Center in Los Angeles, Kathleen Jenkins and Gerardo Marti (2012) discovered “‘older’ (40+) women embracing a ministry that requires them to be wise examples for their younger peers. But their redefinition of ‘being old’ retains aspirations defined by commercial images of physical attractiveness. These women believe they must ‘stay focused on looking fashionable in a healthy body as this is what allows them to prosper in the world in personal relationships and evangelical, missionary outreach’” (quoted in Guest forthcoming). In a similar vein, the ICF’s Ladies Lounge as advertised on the event’s landing page is a prime example for the reproduction of a binary gender distinction by drawing from popular culture, and the forms of femininity represented therein can be analytically separated into an explicit and an implicit level. On an explicit level, as we shall see, women are depicted as sensually attractive and self-confidently professional; on an implicit level, they are relegated to an exclusively female sphere within (but not beyond) which they wield authority and influence. Femininity, in other words, is presented as self-empowering, but only within a specific, postfeminist framwork, one that does not encroach upon the male sphere of authority.

Studies on gender and Evangelicalism confirm this very dichotomy, but have emphasized the ways in which women in conservative Evangelicalism use traditional gender roles as a basis for self-enhancement and self-empowerment. While conservative Evangelicals discursively support traditional gender roles and the submission of wives to their husbands, research concludes that these norms are often impossible to uphold on a practical, day-to-day level: “Evangelical couples’ everyday experiences tend to resemble ‘mutual’ submission in which husbands and wives share household responsibilities. […] Women often work outside of the home and men help out with childcare and domestic tasks […] in order to sustain a middle-class lifestyle” thereby being able “to call themselves anti-feminist while taking advantage of material advantages demanded by the feminist movement, such as women working outside the home” (Burke and McDowell 2012, p. 72). In this way, while formally recognizing female submission, women performatively and functionally claim influence over family decisions (e.g., Ammerman 1987) and a degree of autonomy and control over their own lives (e.g., Griffith 1997), while men are increasingly involved in the sphere of the home^8^ (e.g. Martin 1998). As Woodhead (2003, p. 77) notes, women thus succeed in “exercis[ing] considerable power, even though this power becomes invisible from a masculine point of view for which ‘real’ power is confined to the ‘public’ realm of the state and market.”

In the context of the analysis to follow, the point of female empowerment through women’s engagement in conservative Evangelical communities, as paradoxical as it may seem at first glance, is decisive. While anti-feminist stances are clearly visible, Evangelicalism has undergone what Aune (2008, p. 281) calls “a kind of internal secularization, whereby, despite conservative ideals, ‘secular’ values of equality have taken root in conservative religion, bringing transformations in gender roles that work favourably for women.” Aune rightly emphasizes that “recent analyses have taken seriously the need to recognize women’s voices and agency rather than dismissing them as victims of false consciousness who collude in their own oppression,” pointing out that one arena of female empowerment is the “parallel world of women’s ministry groups, where they gain leadership opportunities denied to them in their mixed-gender congregations, as well as emotional support from other women” (2008, p. 282). It is this theoretical vantage point—postfeminist discourses in the larger context of self-empowerment in Evangelical women’s ministry—which the analysis of the ICF’s Ladies Lounge 2021 website is based on.

## Methodology: analyzing religious media representations

Any study of the intersections of religion and digital media must take into account perspectives of mediatization, a theoretical approach developed within the past decade to conceptualize the impact of media on religious and broader social changes (Lövheim 2014; Hepp et al. 2010; Hjarvard 2013). In this framework, media—and particularly digital media—are understood as fundamentally shaping religious organizations and practices on the macro, meso, and micro levels, thus effecting social and cultural transformations over time (Lövheim and Hjarvard 2019). According to Lövheim and Hjarvard (2019, p. 213), two aspects are crucial in this context: “a reconfiguration of the relationships between media, religion and other societal domains with regard to influence in society,” and “changes in the conditions of communication and interaction” (Lövheim and Hjarvard 2019, p. 213). Knoblauch, too, has pointed out the central importance of mediatization in the context of popular religion (2009, pp. 210–227). The websites analyzed in the next section must thus be viewed from the perspective of how understandings of femininity are mediatized and symbolically embedded in the religious context of the ICF with transformative effects on how women are culturally represented. Campbell and Evolvi (2019) note that the strong presence of the Internet in everyday life impacts religious identity negotiations, while Lövheim (2012) shows that the Internet may intensify identity performance by providing space to represent one’s beliefs and worldviews while connecting with like-minded others. The analysis that follows focuses on the landing page of the Ladies Lounge 2021 website as a media product, and thus on the “habitus and mode of […] experience of the producers” (Bohnsack 2010, p. 274; our translation), i.e., those individuals involved structurally and/or semantically in the site’s creation and maintenance.

If the focus lies on the websites as media products, then the question of how to analyze media representations must inevitably be addressed. To do so, we draw from Stuart Hall’s seminal work *Representation: Cultural Representations and Signifying Practices* (1997), in which he analyzes explicit and implicit power hierarchies. While Hall’s approach to representation focuses on disclosing practices of racialization by analyzing photographs and their corresponding captions, his approach is methodologically instructive for the present purposes to disclose postfeminist stereotypes of femininity in images and text on the Ladies Lounge 2021 landing page. In analyzing images, Hall differentiates between the scene an image depicts, or the “denotation,” and the meaning it conveys, or the “connotation” (1997, p. 229). Following Roland Barthes (1977), he sees the meaning, or connotation, as revealed “in the conjunction of image and text. Two discourses—the discourse of written language *and* the discourse of photography—are required to produce and ‘fix’ the meaning” (Hall 1997, 228; emphasis in original). Furthermore, Hall argues that meaning always becomes revealed by comparison, depending on the difference between opposites to be intelligible. In the present example, as we will see, femininity becomes comprehensible by being contrasted to, and thus defined by, what it is not, i.e., masculinity. In a similar vein, Woodhead has contrasted what she calls “marked” femininity with “unmarked” masculinity (Woodhead 2007, p. 573). Hall’s thoughts on stereotyping are particularly insightful in this connection: “Stereotyping reduces, essentializes, naturalizes and fixes ‘difference’. […] It symbolically fixes boundaries and excludes everything which does not belong. [And] stereotyping tends to occur where there are gross inequalities of power” (1997, p. 358). The Ladies Lounge 2021 website, as we will argue in what follows, presents a postfeminist version of femininity as normative, drawing from popular culture to mediatize this stereotype and embed it in the ICF’s religious worldview.

**Fig. 1:**
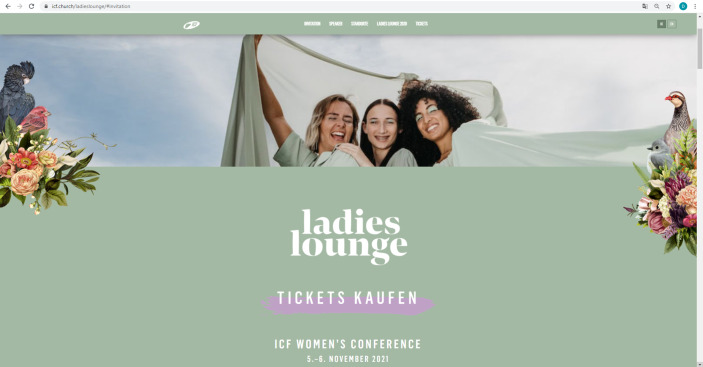
Segment of Ladies Lounge 2021 landing page

**Fig. 2:**
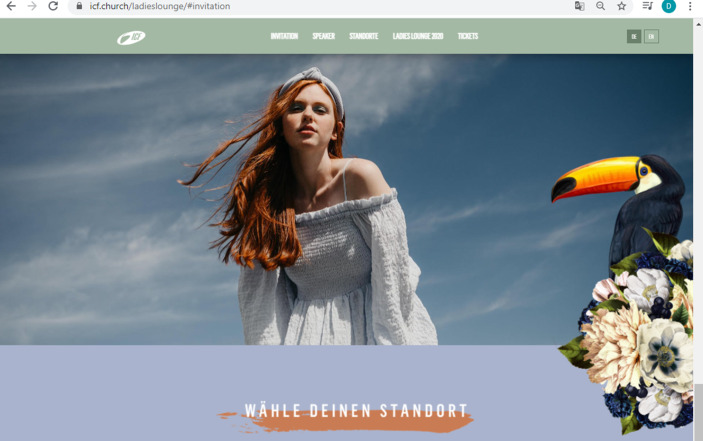
Segment of Ladies Lounge 2021 landing page

**Fig. 3:**
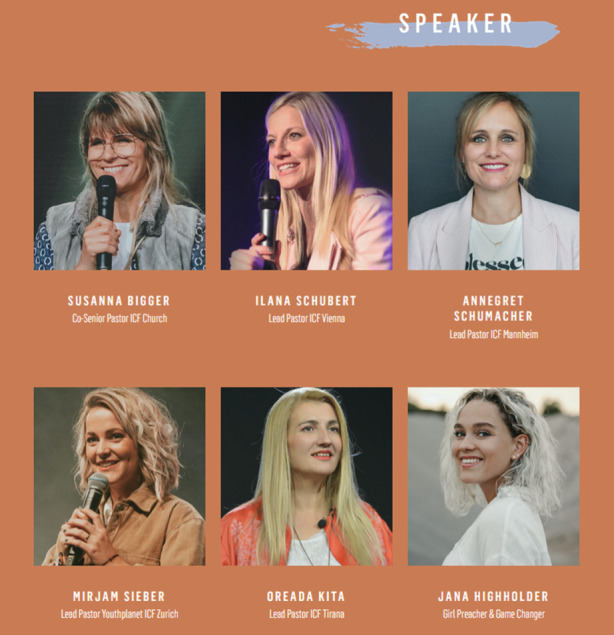
Segment of Ladies Lounge 2021 landing page

In the following section, we analyze three screenshots taken from the Ladies Lounge 2021 landing page^9^. The website is hosted by the International Christian Fellowship and is geared towards female viewers (and, by extension, female event participants^10^). We have chosen this webpage because it advertises the largest event the ICF offers for women and because as such, it reveals dominant notions of femininity as represented online in the ICF in both images and text. We particularly focus on the imagery used on the landing page due to its prominent positioning: the way the various images are arranged on the landing page arguably captures the viewer’s attention more immediately than the single paragraph of text on the landing page does. Despite the importance of text transporting religious notions and convictions, we consider images and photos crucial as strategic and critical media which communicate meaning that goes beyond mere depiction. Images are not only an instrument used to display people, scenes, or ideas, but wield influence through their visuality, symbolism, and semantics (Hall 1997; Morgan 2018). This is especially the case with websites and other digital media, where images are used as part of a visual set-up including graphics, texts, and hyperlinks. While images are often included as one part but not as the center of analyses in the sociology of religion, with this contribution we aim at working towards filling this gap (for more about the importance of images, see, e.g., Vásquez 2011). While we include the landing page’s paragraph of text in the final step of our analysis, we focus first and foremost on analyzing the three screenshots (Figs. 1, 2 and 3) taken from the landing page and extending the results to the page’s other images. For reasons of scope, we do not include an analysis of the video in this article (the audio track of which narrates the paragraph of text we include in the analysis). Readers are advised to visit the website in order to get an overall impression that goes beyond the figures provided below.

For the analysis, we draw from Ulrich Oevermann’s methodological works on objective hermeneutics, which are widely debated in German sociology. Taking as a starting point the sociology of knowledge, Oevermann and his colleagues established objective hermeneutics with the main goal of revealing immanent structures of meaning in text-based material. Objective in the sense of this approach refers to a controlled, step-by-step analysis that generates understanding of the material. Since scholars tend to mistake this methodology as claiming to find an objective truth, it is important to state that the term “objective” is rather used to underline the endeavor of avoiding subjective interpretations. This is achieved by means of constant reflection and joint analysis in a group of researchers, which provides more perspectives and hypotheses in the analytical process and prevents hasty conclusions (see Radermacher 2020, pp. 118–121, for a further discussion on this methodology; see also Wernet 2000). Oevermann later conferred this type of analysis to visual material. In the most basic terms, the raw data is central to the analysis and the approach demands that the researchers temporarily blend out any contextual knowledge. In this way, the material is studied more intensively in its own right, as it were. In reference to visual material, Oevermann states that images already contain a myriad of information in and of themselves, and once the imagery is connected with contextual knowledge, the images become “vessels of knowledge” (Oevermann 2014, p. 36; see also Wernet 2014).

Gregor Betz and Babette Kirchner (2016) used Oevermann’s methodological considerations to establish the *Sequenzanalytische Bildhermeneutik* (sequence analytical image hermeneutics, hereafter visual sequence analysis). Here, the focus is on the visual material itself, and the analysis operates on similar assumptions as Oevermann’s text-based analysis. Betz and Kirchner systematically base their approach on the sequential nature of sociological understanding by proposing three steps for the analysis: (1) the segmentation of the visual material into segments (or sequences) for an irritation of the viewer’s gaze^11^; (2) the sequential hermeneutical reconstruction of the visual material by developing different modes of viewing, or *Seharten *(in this step, other methods can be added to the analysis); and (3) the further interpretation of the image’s contextual function and meaning by taking into consideration its use and context (Betz and Kirchner 2016, p. 263). Again following Oevermann, they posit that the textual and visual sequence analysis is to be conducted in a group of scholars from diverse disciplines to broaden the range of interpretative knowledge and handling of visual data. The scholars are to gather and discuss associations, ideas, and emotions evoked by the visual data, ignoring their contextual knowledge as much as possible during the course of the analysis. Ideally, only the leading researcher herself is aware of the context and knows what the image to be analyzed contains in its entirety. Prior to the group analysis, she divides the image into various segments, initially covering all but the least expressive segment from view. This allows approaching the visual material sequence by sequence and thus deducing the image’s formal structure step by step from within the material^12^. Betz and Kirchner recognize the difficulty of the task of segmentation, because it inevitably requires a subjective decision on part of the researcher regarding not only into which segments to divide the image but also ordering the segments from ‘most boring’ (i.e., least expressive) to ‘catches the eye immediately’ (i.e., most expressive). While the segmentation mostly serves the goal of slowing down the analysis, in the last step of the procedure—analyzing the full image in its context—the different modes of viewing are supplemented with case-specific knowledge. The emphasis here is not on the ‘objectively correct’ segmentation, since this method does not seek to discover an objective truth, but on the constant reflection of each element in reference to the research interest (Betz and Kirchner 2016, pp. 264–67).

There is a range of methods to analyze visual, textual, and audio material in the social sciences (see Flick 2014 for a comprehensive overview; see also e.g., Tsuria et al. 2017; Meier 2016; Hewson 2015; Maeder 2014; Hesse-Bieber and Griffin 2013; Bohnsack 2010; Kim and Kuljis 2010; Schnettler and Raab 2008; Stöckl 2004). In the context of the case study at hand, we worked with a combination of visual and textual sequence analysis because it provides scholars with a methodological instrument that takes into account the full webpage with its various visual and textual elements step-by-step. By avoiding immediately focusing on the symbolic language of images or semantically loaded terms at a first glance, this approach allows for a slow, systematic, and overarching analysis and thereby reconstructs social meaning that goes beyond what is depicted or written. Instead of lengthy descriptions and translations of the imagery into words, the imagery as part of the website composition is investigated in reference to societal structures of meaning. The intermix of sequentiality, irritating the viewer’s gaze, joint interpretation in the analysis group, and contextual framing at the end of the process leads to a broad and multimodal analysis that takes into consideration the complex layering of websites as natural protocols of popular cultural phenomena, in our case within the religious sphere.

The analysis of the Ladies Lounge 2021 landing page below was conducted with a changing group of researchers from different disciplines, including sociology, art history, study of religion, and Japanese studies. We focused on the three screenshots mentioned above, ultimately applying the visual sequence analysis results to the remaining images on the landing page. We simultaneously analyzed textual elements where they appeared in the screenshots (titles, headers, etc.) using textual sequence analysis as sketched above. Below, we present a condensed summary of the research results, as a full record of the case structure generalization (i.e., a discussion of various modes of viewing) would expand the scope of this paper.^13^

## Analysis: representations of femininity on the Ladies Lounge 2021 landing page

If “gender is an effect of language” and “language [is] understood as all forms of representation including, but not limited to, image, gesture, and narrative of all kinds,” as gender studies scholar Darlene Juschka argues (2016, p. 142), then how is femininity represented on the Ladies Lounge 2021 landing page? The results of the group analysis reveal that through its imagery and composition, the website communicates womanhood as linked to two central themes: femininity is associated with *nature, a stylized yet modest concept of beauty, sensuality, *and* shelter*, on the one hand, and with *competence, leadership, self-confidence, *and* professionalism*, on the other. Both themes additionally address the tension between *inclusivity *and* exclusivity* as a core characteristic of femininity in the Ladies Lounge. In this section, we examine these aspects in turn and relate them to the discussions on postfeminism, Evangelicalism and gender, and the mediatization of religion and popular culture sketched above.

Regarding the first theme, the connection between femininity and *nature* is drawn through the use of specific colors and various other elements. Earth colors, such as green (Fig. 1) and orange (Fig. 3), make up most of the images’ backgrounds and reoccur in the shades of the women’s clothing (Fig. 1). The sky is clearly visible in the background of Figs. 1 and 2, suggesting freedom, joy, and space to grow, while the framing earth colors suggest grounding and fertility. The flowers and birds that border the layout of two images (Figs. 1 and 2) emphasize the contrast between earth and sky, grounding and growing, as it were. While the women are depicted in the center as dynamic and open, the flowers in the bordering elements are accurately arranged in neat bouquets, while the birds are not depicted in flight but as sitting unmovingly on top of the flowers. They are seemingly tamed and tranquil, in juxtaposition to the women laughing with outstretched arms in the center. The style of the floral arrangement resembles baroque still life art, which symbolizes the idea of capturing beauty through imagery (for more on the depiction of flowers in art, see, e.g., Uchtmann 2011). This *stylized yet modest idea of beauty* reoccurs in all three images: all women depicted are well-groomed, with (mostly) subtle make-up, carefully arranged hairstyles (even in Fig. 2, where the wind gently blows the woman’s hair away from her face), white teeth (with the exception of the center woman in Fig. 1, who appears to be wearing braces—possibly to symbolize ‘imperfection’ as accepted if there is a willingness to develop into ‘more’), and perfectly arranged clothing. This kind of representation inevitably hints at female perfection and ‘purity’, which is of course a main gender marker and goal for women to strive towards in conservative Evangelicalism (e.g., Gill 2007; Griffith 1997). The plain white font used in all three images, capitalized and without serifs, mirrors the striving towards purity and perfection.

In a parallel vein, femininity is depicted as sensual, joyous, and soft in the images. This is emphasized by the flowing lines of the large, semi-transparent sheet floating in the wind in Fig. 1, and the wind blowing through the hair of the woman depicted in Fig. 2. In both images, the women wear thin, flowing clothes in light colors (light green in Fig. 1, white in Fig. 2). The facial expression in Fig. 2, particularly, communicates the idea of *sensuality*. The red-haired woman is looking down at the viewer from above, seemingly inviting her (the viewer) to join her in feeling the sun and wind on her face and shoulders. Sensuality is represented in a slightly different way in Fig. 1, where the three women stand close together under the shelter of the sheet, creating the image of a close-knit community characterized by laughter and joy. In all three images, no negative or overly emotional outbursts are depicted in the facial expressions and body language of the women; instead, viewers arguably get an impression of peace, comfort, and friendship. This brings to mind the idea of “being oneself” and “pleasing oneself” as a central marker of postfeminist thought (Gill 2007, p. 153): The women depicted seem to be entranced by the beauty of the sunshine, the sensuality of the wind, the intimacy of female community, themes which all resonate with the Christian story of creation; and as such, they seem liberated from inequality and oppression. This representation of femininity inevitably completely disregards its own social construction and mass-mediatization.

Importantly, the large and semi-transparent sheet in Fig. 1 represents a *shelter* of sorts, seemingly offering an intimate space of female community. A safe space, a nurturing environment is represented here, and the viewer is invited into the fold by way of the link “*TICKETS KAUFEN*” (“Buy tickets”) in the bottom third of the image, underscored in light mauve. The imagery is reminiscent of (originally medieval) depictions of the Virgin Mary as *Schutzmantelmadonna,* the Virgin of Mercy, offering protection to women, children, and the wounded under her cloak (see e.g., Rothes [1909] 2013). The three women under the cloth offer the viewer a momentary glace at the shelter and community offered by the Ladies Lounge; the photograph is arguably choreographed in such way as to encourage the viewer to want to find out more, and quick, before the window of opportunity to join closes. Shelter and community are available, but only if you act fast—and, crucially, only if you fit (i.e., conform to) the kind of femininity dominant in the Ladies Lounge. This representation of femininity arguably directly connects to gender roles constructed in the course of the nineteenth century that cast women as responsible for the home and hearth (e.g., Winkel 2018; see also Guest forthcoming).

At a first glance, it seems that all women are invited to join the Ladies Lounge and to metaphorically find shelter under the sheet by joining the community. This seeming inclusivity is represented in the slightly diverse depictions of the women in all images: there is a blond, a brunette, and a woman with brown skin and curly black hair in Fig. 1, a redhead in Fig. 2, and a range of ages represented in the photographs in Fig. 3. But at a second glance, the women do not represent a wide variety of body types, styles, ages, or skin tones at all. They all appear healthy, slim, and fit, seem to be between 20 and 45 years of age, and their skin color is mostly white or light brown. Femininity is thus discursively limited to being young, healthy, attractive, and light-skinned, which excludes older women, women with disabilities, dark brown and black women, women considered ‘unattractive’ by the beauty standards communicated via mass media and popular culture, and women otherwise deviating from the norms set on the Ladies Lounge 2021 landing page. As shown above, this conception of femininity is also emphasized in the elements depicting the birds and floral arrangements that border Figs. 1 and 2: the birds all look different—the toucan in Fig. 2 may even be described as ‘exotic’—but they sit among the orderly arranged flowers and stare about demurely. They are pretty to look at but do not represent activity or engagement. Instead, they link femininity in the Ladies Lounge with tradition, both in terms of appearance and sphere of action, thereby suggesting that beauty and tranquility characterize womanhood in the Ladies Lounge, assigning women to the sphere of the home, of shelter, nurture, and domestication. This resonates with McRobbie’s assessment that postfeminism harbors conservative ideals behind the use of modern styles (McRobbie 2004). However, this reading is complicated somewhat when we cast a closer look at Fig. 3, which serves a different function on the webpage and opens up the second theme in our research results.

The depictions of the Ladies Lounge 2021 speakers in Fig. 3 suggest that femininity at ICF is not only associated with the private, but also with the public sphere, particularly with professional *competence* and *leadership*. The women shown all hold leadership positions, at least according to their titles: the ICF’s co-senior pastor, several lead pastors of various ICF congregations in Switzerland, Austria, Germany, and even Albania, and two youth leaders are depicted. This suggests not only that the ICF is a large organization, extending beyond the German-speaking countries of western and central Europe into eastern Europe, but that women have the opportunity of actively participating in and shaping the church. The speakers are presented as authority figures who teach, lead, and inspire other women to strive for more. What for exactly, we do not know, because there are no references in the analyzed images (or, interestingly, on the landing page as a whole) as to what specific content the Ladies Lounge 2021 actually focuses on. The speakers’ authority is not only communicated via their titles, but inevitably also and crucially through their photographic depictions: All six women communicate *self-confidence* and *professionalism* by way of their straight body postures and their direct and open gazes, whether they are looking at the viewer or not. Three of them hold microphones, suggesting they are speaking to large audiences in technologically well-equipped settings, visually highlighting the fact that they are established professionals in the organization. These representations are related to individual religiosity in the sense that each woman has ‘her own voice’ with which to vocalize her religious convictions.

Closer scrutiny, however, reveals parallels with the representations of femininity discussed above. There are no strong, expressive gestures visible; the women’s arms hang by their sides unless they are holding a microphone, which all three hold close to their torsos (instead of, e.g., away from their bodies to raise their voices). Their expressions are friendly, open, and welcoming; there is no anger or stronger emotion of other sorts visible. In parallel to the other two images analyzed, all women in Fig. 3 wear light clothing as at least part of their attire and smile openly, displaying perfect teeth. Their make-up is subtle and all six are blondes, with a range from light blond to dyed blond. Again, diversity is essentially not displayed, or only on a very limited scale. Feminine leadership and professionalism are represented here as simultaneously proactive and restrained; five of the six women are smiling at the camera in what seem to be posed photos, while only one is shown actually interacting with her audience (she seems to be speaking while gesturing with her left hand). In this way, representations of femininity as well-groomed but modest, as sensual instead of assertive are again reproduced.

At the same time, however ‘soft’ female leadership at the Ladies Lounge is made out to be in this image, it is worth noting that the fact that women are able to take on such visible and vocal leadership roles at the ICF is striking and not at all typical for conservative Evangelicalism. This view supports Aune’s (2008) argument that women’s ministry groups function to empower Evangelical women, giving them agency and the opportunity to extend emotional support to others. In this way, they are able to claim leadership within a limited sphere—in the case of the ICF, the Ladies Lounge—and shape this sphere as they see fit. While the speakers do not seem to be advocating a total transformation of gender roles, they are using the influence they have to empower women to organize. For what, again, we do not know; we can assume that this is no fourth-wave feminist movement, but rather clearly an event centered around postfeminist religious values. These women seem set on reclaiming ‘original’ and ‘authentic’ femininity in the way they are presented here and at the same time on combining private and public life by taking on leadership roles in professional and self-confident ways, thus conforming to contemporary conservative Evangelical understandings of gender (see the theoretical section above).

Susanna Bigger’s title “Co-Senior Pastor ICF Church” (Fig. 3) briefly deserves a closer look. Bigger shares the title of senior pastor with her husband Leo. Co-pastor arrangements frequently occur in Evangelicalism, typically with a married couple sharing the position to simultaneously communicate equality of the sexes and a traditional, heteronormative family model, again suggesting a tension between female emancipation and submission (see Burke and McDowell 2012). Women in the ICF, in other words, have the opportunity to advance to the top rungs of the organizational hierarchy but are always backed by male counterparts. A more detailed discussion of this topic is beyond the scope of the present paper but deserves a brief mention, as our analysis group zoomed in on the term “co-pastor” in one discussion.

A central theme visible in all analyzed images is that of *inclusivity* vs. *exclusivity*. As discussed above, at first glance the Ladies Lounge presents itself as welcoming all women. Closer scrutiny reveals that the types of women depicted in the images represent only a very narrow understanding of femininity, namely as young, slim, healthy, attractive, modest, and so forth. In this sense, the Ladies Lounge excludes women who deviate from these norms. What is more, the Ladies Lounge is arguably milieu-specific in that it addresses comparatively well-off, ‘middle class’ women who feel comfortable enough with ‘lounge’ associations, including an exclusive get-together, a special clientele, and specific rules of interaction, but also approachability and a degree of informality and comfort. The Ladies Lounge 2021 is represented neither as an academic conference nor as a self-help meeting, but as an extraordinary event catering to a certain type of woman. The fact that the landing page does not reveal what exactly the Ladies Lounge is about arguably encourages the viewers to delve more deeply into the website to gather more information and ultimately buy a ticket. This again suggests exclusivity: you must buy a ticket to become part of the crowd, to join the shelter of the community; and should the number of tickets be limited, you should reserve your own ticket quickly. The market logic is especially salient in the central position of the “Buy tickets” link in Fig. 1: it is positioned immediately underneath the event title and set off against the pale green background by the light mauve highlighting. Underneath the stylish veneer, a basic economic transaction is waiting to be made: secure a ticket to buy your way in and become part of the in-crowd; invest in your standing to boost your status in the group (on the role of market logics in religion, see, e.g., Einstein 2008; Gauthier and Martikainen 2013). The popular cultural aesthetics the landing page as a whole is steeped in become very apparent here in particular: The user could be buying event tickets (or checking out different locations, as suggested in the link *WÄHLE DEINEN STANDORT*—“choose your location”—in Fig. 2) for a secular concert or show, or a similar cultural event; there is no reference in Fig. 1 or 2 that the Ladies Lounge is embedded in a religious context.

Taken together, the visual sequence analysis reveals that femininity on the Ladies Lounge 2021 landing page is represented as sensual, sheltered, and as conforming to popular cultural standards of modesty and beauty alike, on the one hand, and as self-confident and professional, as embodying leadership and competence, on the other. The women-only event format suggests that female leadership is emphasized especially within the realm of women’s ministry, and the representations of female leaders on the landing page connote women’s empowerment as much as postfeminist restraint from asserting authority beyond the ‘female sphere’. To the viewer, the landing page as a whole, as a media representation of the Ladies Lounge 2021, communicates the event (and the women involved) as modern and emancipated, as a space for women to connect ‘as they are’, ‘naturally’, unpretentiously. A closer look reveals the conservative undercurrents in this representation of femininity: beauty ideals mirroring the entertainment industry, very little diversity, and a reserved style that expresses gentle emotions both in the private realm (represented by the women depicted outside) and the professional realm (represented by the pictures of the speakers/leaders). It is important at this point to note that the only religious references that appear in the images on the landing page are the leaders’ titles (“pastor” or “preacher”) and the co-senior pastor’s affiliation (“ICF church”). For this reason, while we were able to include these minor references to the Ladies Lounge’s religious context in the first steps of the sequence analysis, we incorporated the religious context more broadly in the final step of the analysis by examining the only longer section of text on the landing page.

In this text, the representations of femininity analyzed in the images are reproduced, highlighted, and framed from a biblical perspective. Titled “one voice”, it extends an invitation to readers to attend the Ladies Lounge 2021 as part of a diverse group of women who come together to find a “common voice” to “stand up for […] God”^14^. Every woman’s (i.e., future participant’s) uniqueness is addressed (“Each one of us is different and we are uniquely created with our own thoughts and words. Some louder, some quieter, some colorful, some tone in tone”), while a shared space of belonging is discursively created by framing the communal experience of the divine and intimately confiding in God as the basis of the Ladies Lounge (“God gives us the possibility to come together so that we can experience something great. […] With one voice we raise his name [and] pour out our heart to him”). The supposed diversity of the women coming together is juxtaposed by the way in which it is immediately narrowed and channeled into one and the same “voice”; the event’s female community is, in fact, represented as uniform instead of diverse, as homogenous instead of plural, when the text states that “We are one unity and that is our strength. With one heart. Undivided. With Jesus”. What is more, the fact that women at the Ladies Lounge “pour out our heart” to God and stand as one “with Jesus” suggests female submission to male authority figures^19^, inevitably reproducing heteronormative gender roles in the very act of claiming agency to worship God as a group of empowered women. A Bible verse from Romans is quoted that frames God as a source of “great endurance,” “comfort,” and “grace,” and foresees his followers (i.e., participants of the Ladies Lounge 2021) to “glorify God” in “a unanimous rush of passion.” Female submission to God is thus framed as emotional, even passionate, with what may be interpreted as sexual undertones not uncommon in Evangelical framings of women’s relationships to God or Jesus (e.g., Luhrmann 2012). At the same time, God is depicted as “the Father of our Lord Jesus Christ,” evoking the sphere of the home and family, typically attributed to femininity, as we have shown above.

The text “one voice” provides a clearly religious context to the Ladies Lounge event. It is methodologically insightful—i.e., it confirms the methodology’s intent and applicability—that an incorporation of the text’s semantics into the analysis in its final stage does not reveal a new mode of reading how femininity is represented on the landing page. Instead, it confirms the previously distilled modes of viewing and seeing by connoting femininity as supposedly diverse, professional, and open to taking on leadership but ultimately as uniform, connecting it to sensuality, emotion, and nurturing, and confining its sphere of influence to women’s ministry.

## Concluding remarks

In this contribution, we have argued that understandings of femininity as represented on the Ladies Lounge 2021 landing page are strongly influenced by postfeminist discourses and values, communicating conservative understandings of womanhood and ultimately reproducing the heteronormative gender binary in a modern design. While women are depicted as competent professionals and self-confident leaders, they are nevertheless relegated to a specific sphere, that of women’s ministry, to embody these values. At the same time, femininity is depicted as sensual, emotional, and nurturing, further highlighting traditional gender roles at work in the ICF. The images analyzed above (and other, very similar images on the landing page) are strongly reminiscent of the male gaze:

In a world ordered by sexual imbalance, pleasure in looking has been split between active/male and passive/female. The determining male gaze projects its fantasy on to the female figure which is styled accordingly. In their traditional exhibitionist role, women are simultaneously looked at and displayed, with their appearance coded for strong visual and erotic impact so that they can be said to connote *to-be-looked-at-ness*. (Mulvey 1999, p. 837)

While the Ladies Lounge landing page is not designed primarily for male viewers, of course, the male gaze nevertheless plays a crucial role in how femininity is presented here. Women are on display as points of orientation for other women to communicate what femininity supposedly ‘is’: a well-groomed, modest yet ‘naturally’ beautiful appearance; being young and healthy; sensuality and ‘soft’ emotions; asserting leadership gently and with restraint; claiming expertise in a sphere one is intimately acquainted with by default, i.e., what it means to be a Christian woman. Waiting for God to “grace” them and arouse their passion, the women depicted are very aware of their ‘*being-looked-at-ness’. *Mediatized representations of femininity in the Ladies Lounge arguably connote what ‘ideal womanhood’ implies in the religious worldview of the ICF. As can be seen in such websites, the religious and the popular cultural spheres have become deeply entwined in the process of religious marking (*Markierung*) by way of drawing from popular culture to transport religious messages. In this sense, the Ladies Lounge’s use of popular culture to reinforce religious perspectives presents more than a mere blurring of boundaries (*Entgrenzung*) between the two spheres but rather a subtle, but crucial, emphasis on conservative social values which shape the ICF’s worldview as a religious community. It is in this way that new meanings emerge at the intersection of what Forbes and Mayhem call “religion and popular culture in dialogue” (2000, p. 10).

That said, we want to emphasize once more “the need to recognize women’s voices and agency rather than dismissing them as victims of false consciousness who collude in their own oppression” (Aune 2008, p. 282). Our analysis shows that women have the opportunity to claim leadership and exert authority in the Ladies Lounge, empowering other women to gain agency in different contexts. While gender-segregated Evangelical conferences often reproduce the traditional gender binary in very direct ways—e.g., by suggesting women should diet, exercise, get make-overs and even plastic surgery to meet beauty standards of the mass-mediated entertainment industry (e.g., Maddox 2013)—the Ladies Lounge is more subtle in its mediatization of ideal womanhood. Our analysis has shown that femininity is not sexualized in the ICF’s post-feminist interpretation but cast in a beautiful-yet-modest frame. More than undergoing what Aune has called “internal secularization” (Aune 2008, p. 281), the ICF redefines the female sphere of action and authority from a religious perspective. At the same time, the way that Ladies Lounge leaders embody femininity reproduces heteronormative gender roles in many ways due to the very fact that femininity is represented as sensual and nurturing in the private sphere of female community and as gently restrained in the more public sphere of female professionalism.

By focusing on the dynamics of online representations of femininity and conservative values presented in a modern, popular cultural guise, we have provided an insight into the negotiation processes of women in the Evangelical context and thus contributed to the growing body of research on postfeminism and gender in Evangelicalism. The multilayered make-up of the website also represents women’s complex situations and positions between emancipation and tradition within contemporary Evangelical Christianity. Further studies on postfeminism and gender in these contexts could include a look at the other end of the gender binary, namely at men’s ministry in Evangelical communities. While no men’s ministry event is advertised as broadly as the Ladies Lounge on the ICF’s main website, individual congregations do have men’s groups that arguably construct gender in similar ways (for instance, a past ICF men’s ministry group was called “McGyver”). Analyses of how the male gender is represented in these circles would fruitfully complement the analysis in this article and further contribute to discussions on the mediatization of postfeminism and gender in Evangelicalism.

## Footnotes

1 We define “neo-conservative” broadly as those conservative religious communities that tap into mass (especially social) media, cutting-edge technology, the market logic, and other popular cultural elements, including aesthetics, in order to present themselves to a large audience with a carefully drafted image, using the internet as a crucial space to attract new members.

2 Considering our own research on Vineyard DACH (Deutschland, Österreich, Schweiz), it is important to point out that the ICF has adopted features from Vineyard USA instead of from Vineyard DACH, which differs from its American sister organization in several respects (Freudenberg 2019a, b).

3 https://www.icf.church/en/locations/ (retrieved August 16, 2021).

4 See, for instance, https://www.icf.church/zuerich/en/ladies/#events or https://www.icf.church/zuerich/de/propel-women/ (retrieved August 16, 2021).

5 https://www.icf.church/en/ (retrieved August 16, 2021).

6 McRobbie (2004) rightly criticizes both Beck (1987) and Giddens (1991) for failing to take into account gender inequities in their works.

7 Lanwerd notes that approaches to representation fulfill three crucial functions in the study of religion: 1) They focus on gender relations as representations of religious rule systems; 2) they reflect the value attested to the differences between ‘male’ and ‘female’; and 3) they inquire after the right to define and assess these differences (2008, p. 212).

8 The “domestication” of men via Evangelicalism and Pentecostalism, particularly in Latin America’s *machismo* culture, has produced a number of insightful studies in recent decades (e.g., Martin 1990).

9 https://www.icf.church/ladieslounge/en/ (retrieved August 16, 2021).

10 At the time of finalizing this article, in August 2021, the landing page did not mention possible restrictions to physically attending the event, scheduled for November 2021, due to the Covid-19 pandemic. We mention this here because while the format in which the Ladies Lounge 2021 ends up taking place is irrelevant to the topic at hand, the fact that the landing page included a tab labelled “Standorte” (German for “locations”) sparked a discussion in our analysis group regarding the potential importance of multiple event locations for the broader visibility of the ICF.

11 Textual and visual sequence analysis systematically irritates reading or viewing. Irritating the viewer’s gaze is crucial in this type of analysis because it helps focus on each segment without distractions regarding what may follow. Especially when dealing with visual data, which is often imbued with specific symbolic imagery and indexical references, first impressions of a full image may influence the general perception of the data if not irritated on purpose. Instead of immediately taking into consideration the entirety of the depiction or following the (assumed) intention of an artist, the irritation of the viewer’s gaze serves as a means of equalizing the different segments to avoid biases during the analysis (Betz and Kirchner 2016, pp. 266–67).

12 By uncovering seemingly peripheral sequences of an image, researchers are able to develop different modes of viewing. The group is to gather various distinct modes of viewing during the course of the analysis, as each additionally uncovered segment may facilitate other interpretative suggestions. The further one progresses in the analytical procedure, the more likely it is that modes of viewing are either affirmed or discarded. Ideally, once the analysis is completed, only one mode of viewing remains as the one exposing the image’s immanent structures of meaning (Betz and Kirchner 2016, pp. 267–68).

13 A case structure generalization (*Fallstrukturgeneralisierung*) follows the analytical process and describes the development of different modes of viewing and the formulation of the immanent structures of meaning by constantly referring back to the material and the associations evoked by it (Oevermann et al. 1979).

14 Quotes taken from the text found on the Ladies Lounge 2021 landing page: https://www.icf.church/ladieslounge/en/ (retrieved August 16, 2021).

19 For an extensive discussion on the topic of Evangelicalism and authority, see the special issue on by Freudenberg et al. (forthcoming) on “Religious Authority in Practice in Contemporary Evangelical, Charismatic, and Pentecostal Christianity”.
